# Cerebral amyloid angiopathy-related inflammation (CAA-ri): A case report

**DOI:** 10.1097/MD.0000000000041010

**Published:** 2024-12-20

**Authors:** Yao Meng, Yining Xiao, Ruohan Sun, Lingyu Li, Yanhong Dong

**Affiliations:** a Department of Neurology, Hebei General Hospital, Shijiazhuang, China; b Department of Neurology, Graduate School of Hebei North University, Zhangjiakou, China; c Hebei Provincial Key Laboratory of Cerebral Networks and Cognitive Disorders, Shijiazhuang, China; d Graduate School of Hebei Medical University, Shijiazhuang, China; e Graduate School of North China University of Science and Technology, Tangshan, China.

**Keywords:** β-amyloid protein, cerebral amyloid angiopathy-related inflammation, immunosuppressive therapy

## Abstract

**Rationale:**

Cerebral amyloid angiopathy-related inflammation (CAA-ri) is a treatable condition characterized by an acute or subacute onset, with its primary pathological hallmark being the deposition of amyloid, predominantly β-amyloid (Aβ), within intracranial microvessels. Despite its potential for treatment, CAA-ri is a rare disorder that is frequently underrecognized by clinicians in practice. This article provides a comprehensive overview of the clinical manifestations and therapeutic approaches associated with CAA-ri, aiming to enhance awareness among healthcare professionals.

**Patient concerns:**

A 67-year-old male patient who suffered from a sudden decline in cognitive functioning, intermittent headache, and dysphoria underwent brain magnetic resonance imaging, susceptibility weighted imaging, and cerebrospinal fluid analysis and was considered probable CAA-ri.

**Diagnosis interventions:**

During the course of disease development, the patient suffered from a sudden decline in cognitive functioning, mainly in the form of unresponsiveness, decreased comprehension, and increased repetitive language, accompanied by intermittent headaches and dysphoria. Brain magnetic resonance imaging showed numerous white matter in both hemispheres. Susceptibility weighted imaging showed multiple spots of hypointensity in the bilateral cerebral and cerebellar hemispheres, a hypointensity signal in the left occipital lobe, and extensive zones of hypointensity in bilateral sulci. Cerebrospinal fluid analysis was abnormal with elevated levels of protein and low levels of P-tau, Aβ42, and Aβ1-42/Aβ1-40. The use of glucocorticoids greatly reduced his symptoms. This lends credence to the probable CAA-ri diagnosis. The symptoms can be successfully alleviated by administering methylprednisolone sodium succinate.

**Outcomes:**

During the patient’s hospitalization, immunosuppressive therapy, primarily consisting of methylprednisolone sodium succinate and methylprednisolone, was administered, resulting in a significant improvement in symptoms. Post-discharge, the patient was monitored regularly, revealing a gradual enhancement in cognitive function without recurrence. Consequently, immunosuppressive therapy was discontinued 1 year following the patient’s discharge.

**Lessons:**

CAA-ri is a rare clinical condition, and timely diagnosis and early treatment are very critical for patient prognosis.

## 1. Introduction

Cerebral amyloid angiopathy-related inflammation (CAA-ri) is an inflammatory condition caused by autoimmunity that results from the deposition of β-amyloid protein (Aβ) with acute or subacute cognitive impairment, followed by transient focal neurological episodes (TFNEs), headaches, and seizures. White matter hyperintensity in the T2/fluid-attenuated inversion is the most major neuroradiological finding. Aβ is a small peptide with 39 to 43 amino acid residues generated by endoproteolytic cleavage of the amyloid precursor protein (APP), and excessive Aβ depositions can damage the ability of learning and retention. Early administration of immunosuppressive therapy has the potential to enhance clinical and imaging outcomes and reduce the duration of the disease in individuals with CAA-ri. Consequently, this study presents a case of probable CAA-ri to increase awareness and understanding of this condition.

## 2. Case report

**Figure 1.**
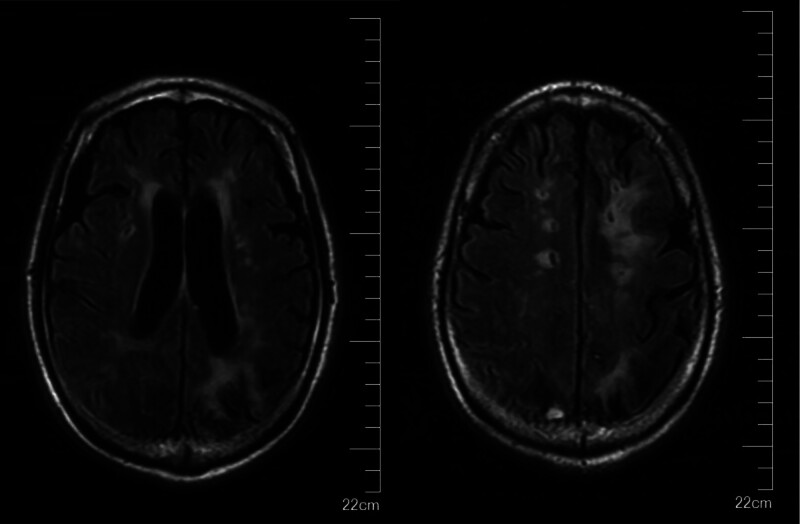
Numerous WMH in the lateral ventricles bilaterally, and the frontal-parietal-occipital-temporal lobes and basal ganglia bilaterally. EPVS in the bilateral cerebral hemispheres. EPVS = enlarged perivascular space, WMH = white matter hyperintensity.

**Figure 2.**
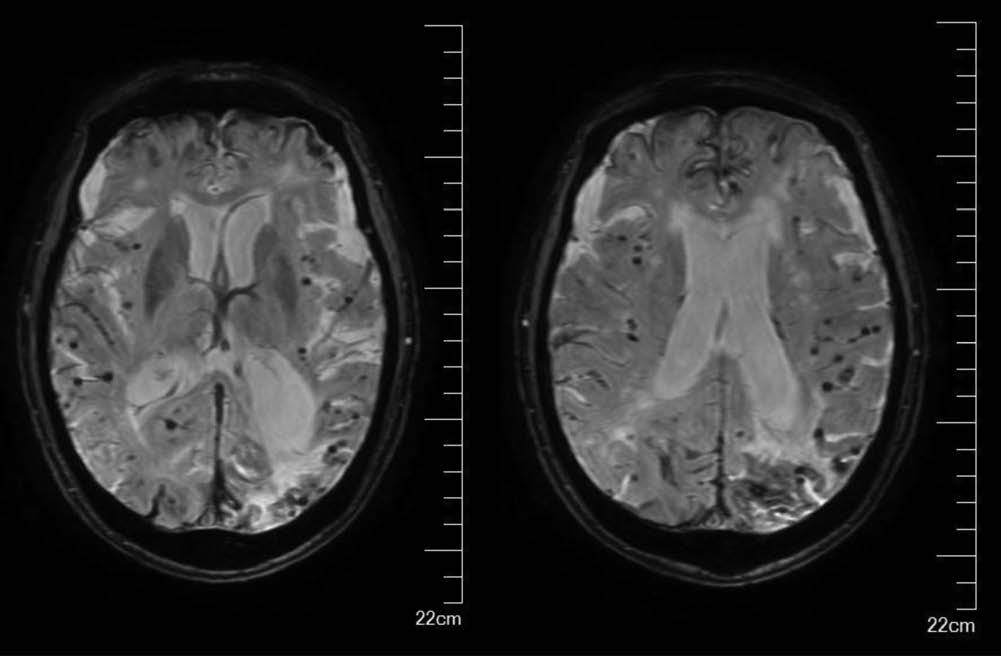
Multiple cMBs were shown on SWI sequence. cMBs = cerebral microbleeds. SWI = susceptibility weighted imaging.

**Figure 3.**
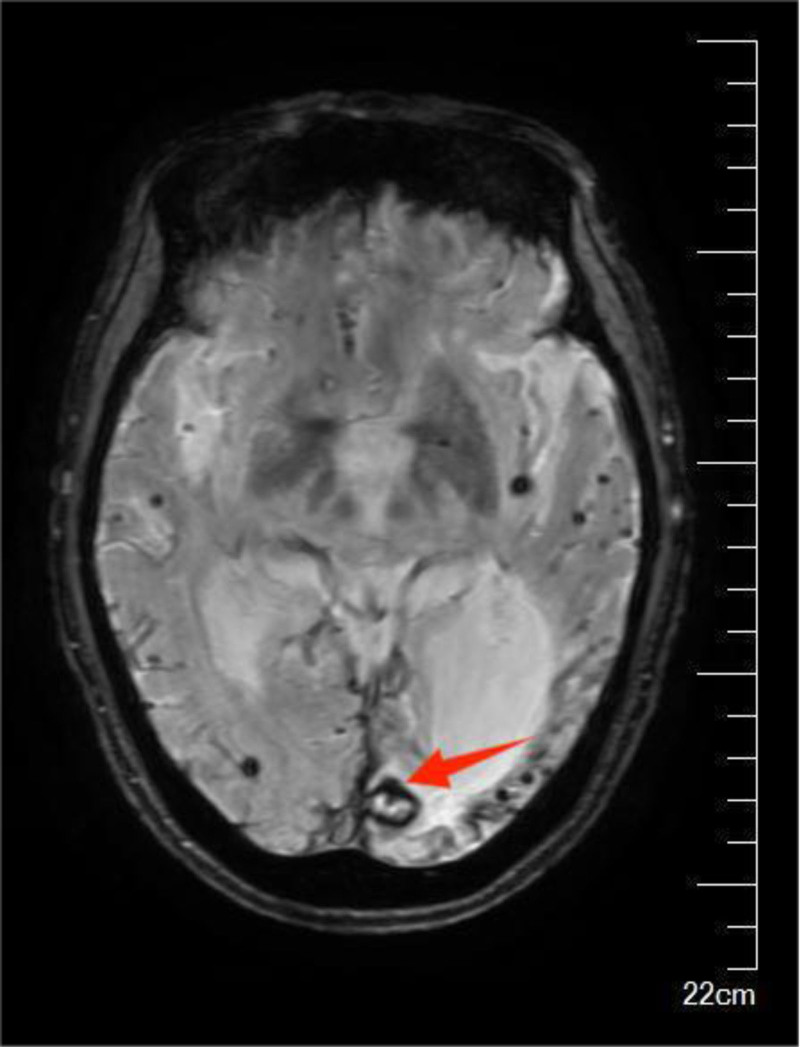
Deposition of accumulated hemosiderin visible on the left occipital lobe lesion.

**Figure 4.**
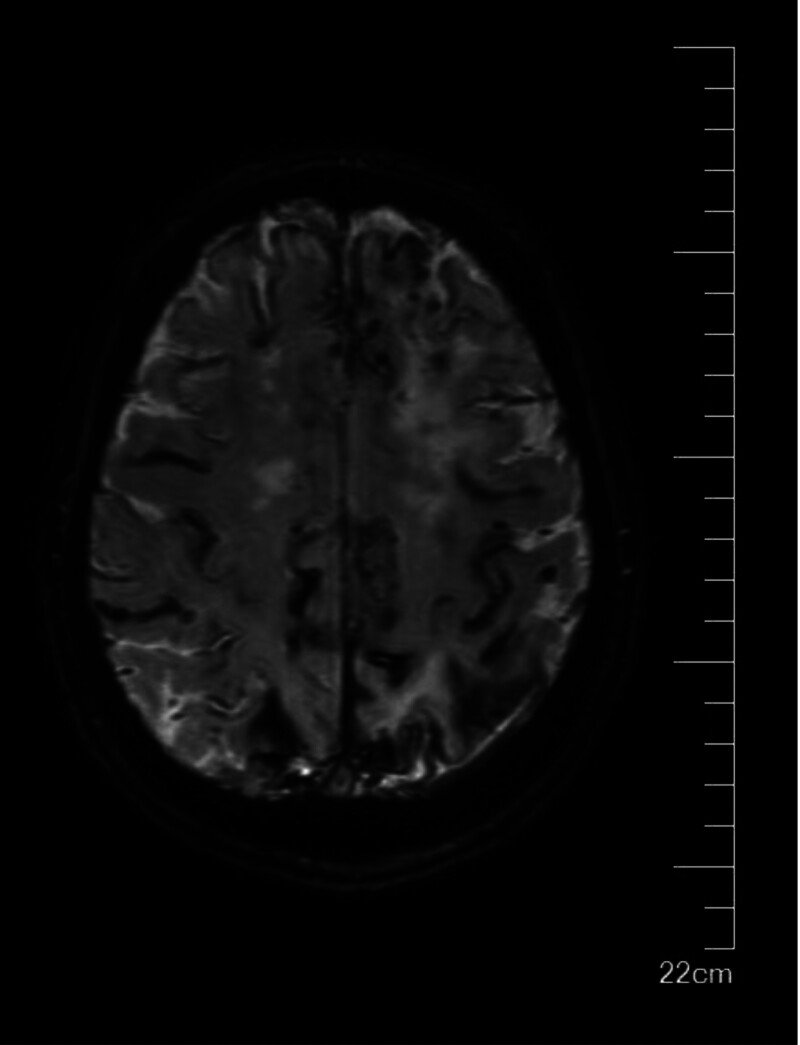
SWI image of dispersed cortical SAH. SAH = subarachnoid hemorrhage. SWI = susceptibility weighted imaging.

A 67-year-old man with a history of lobar intracerebral hemorrhage (ICH) and hyperthyroidism presented with a 5-day history of cognitive decline and intermittent headache. The patient’s condition showed no obvious improvement after pain treatment, and neurological examination revealed no additional neurological signs except the above-mentioned symptoms. There were no abnormal laboratory findings from routine blood samples, serum tumor markers, or immune system examination. Tests for human immunodeficiency virus and treponema pallidum were negative. Upon admission, an magnetic resonance imaging (MRI) of the brain was performed, which showed numerous confluent T2/FLAIR hyperintense areas in the white matter of both hemispheres (Fig. 1). Pronounced on the susceptibility weighted imaging (SWI) image, multiple spots of hypointensity in bilateral cerebral hemispheres and bilateral cerebellar hemispheres (Figs. 2 and 3), and the left occipital lobe coated with hypointensity was also described (Fig. 3). Extensive zones of hypointensity in bilateral sulci were present (Fig. 4). Lumbar puncture revealed normal intracranial pressure, and colorless, clear cerebrospinal fluid (CSF) can be seen. CSF analysis was abnormal with elevated levels of protein (89.02 mg/dL; normal 0–50), T-tau (631.48 pg/mL), and low levels of P-tau (33.21 pg/mL), Aβ 42 (3.14 pg/mL), and Aβ 1-42/Aβ 1-40 (0.08). Scattered fresh erythrocytes were shown in cytologic analysis of CSF. The results of autoimmune encephalitis antibody tests, including the serum and CSF, were normal.

Based on the patient’s clinical course and neuroradiological findings, “cerebral amyloid angiopathy-related inflammation” was first considered. Since admission, the patient had been placed on the edaravone and Xingnaojing (XNJ) injection. Lipid peroxidation, oxidative modification of proteins, and oxidative damage to DNA/RNA are all factors that can impair cognitive function. Edaravone, a free radical scavenger, has been shown to significantly inhibit oxidative damage and neuroinflammation in cerebral white matter, as well as reduce Aβ/p-Tau accumulation, thereby ameliorating cognitive deficits.^[1]^ XNJ injection, derived from the traditional Chinese medicine Angong Niuhuang Pill, serves as a neuroprotective agent. Administration of XNJ has been demonstrated to attenuate memory dysfunction and enhance dendritic spine density.^[2]^ Additionally, the patient received antipsychotic medications for intermittent irritability. Methylprednisolone Sodium Succinate in a dose of 1 g daily was introduced for 5 days, followed by a dose of 80 mg daily. The patient was discharged with an improved neurological condition and remarkable cognitive recovery, with treatment recommendations of oral methylprednisolone 40 mg daily with gradual tapering. The efficacy of immunosuppressive therapy in patients with CAA-ri can be evaluated through imaging changes and symptom alleviation. In this case, the decision to discontinue treatment was primarily informed by the patient’s symptomatic improvement. Following the patient’s discharge from the hospital, a series of follow-up assessments were conducted, revealing a progressive enhancement in cognitive function. The patient demonstrated the ability to independently engage in activities of daily living, including dressing, eating, and oral hygiene, and was capable of composing complete sentences in writing. One year post-discharge, the patient demonstrated enhanced cognitive responses and comprehension than before; he was able to communicate effectively, his repetitive speech had resolved, and he experienced no further headaches or psychiatric symptoms. Consequently, the medication was discontinued 1 year after discharge. It is noteworthy that the patient did not experience a relapse following the cessation of therapy.

## 3. Discussion

Cerebral amyloid angiopathy (CAA) is a progressive cerebral small-vessel disease marked by the deposition of amyloid in the vascular walls of intracranial microvessels, particularly within the arterial walls of the cerebral and cerebellar meninges and cortex.^[3]^ This condition is a significant cause of non-hypertensive cerebral hemorrhage and cognitive decline in the elderly population. The primary clinical manifestations include cognitive decline, seizures, neurological symptoms, and TFNEs. CAA can be categorized into 3 main types: sporadic, hereditary, and iatrogenic, with sporadic CAA being the most prevalent, while iatrogenic CAA is a newly discovered concept.^[4]^ But the pathogenesis of CAA remains unclear. Hereditary forms of CAA are linked to mutations in genes encoding specific proteins or their precursors, primarily amyloid protein, cystatin C, prion protein, transthyretin, and gelsolin. Numerous gene polymorphisms have been identified in association with sporadic CAA or CAA-related hemorrhages, including those in apolipoprotein E (APOE), presenilin 1, and α1-antichymotrypsin. Notably, the APOE alleles ε2 and ε4 are independently correlated with an increased risk of ICH.^[5]^ Furthermore, cerebral amyloid angiopathy (CAA) exhibits a strong correlation with both advancing age and Alzheimer disease (AD).^[6]^ In patients with AD, the administration of immunosuppressive therapy may elevate the risk of developing CAA.^[7]^

CAA-ri is a rare subset of CAA and characterized by deposition of Aβ in the media and adventitia of cortical and leptomeningeal vessels in or around where accompanied by a nondestructive inflammatory reaction. The potential factors predisposing certain patients with CAA to develop CAA-ri remain unclear. The pathogenesis of CAA-ri has yet to be fully elucidated; however, it may be linked to an autoimmune inflammatory response triggered by the abnormal deposition of amyloid protein. Currently, 2 pathological subtypes are widely recognized: nondestructive perivascular inflammation, referred to as inflammatory CAA, and transmural or intramural inflammation, known as Aβ-related angiitis (ABRA). Gadolinium contrast enhancement observed in MRI scans may contribute to the development of ABRA, while the presence of the ApoE-ε4 allele may be associated with inflammatory CAA. Both variants of CAA-ri can manifest concurrently and exhibit similar behaviors. Analyses of previously published biopsy-confirmed cases have identified no correlation between the pathological subtypes and factors such as age at presentation, mode of clinical presentation, response to treatment, or prognosis. However, ABRA may be regarded as a subtype of granulomatous primary angiitis of the central nervous system, which typically necessitates a combination of pharmacological interventions.^[8]^ No definitive clinical or radiological distinguishing features have been found to demonstrate whether ABRA and CAA-ri are 2 separate diseases or represent a point in a single disease process. It is evident that future research should focus on this area. The ApoE-ε4 allele, particularly ε4/ε4 homozygosity, has been recognized as the sole established risk factor for CAA-ri, as previous studies have indicated a high prevalence of ε4/ε4 (76.9%) among patients with CAA-ri.^[9]^ The prominent clinical features are rapidly progressive cognitive decline, focal neurological deficits, encephalopathy, seizures, and headache. Neuroimaging characteristics supporting patients with CAA-ri include white matter lesions, cerebral microbleeds, cortical superficial siderosis (cSS), cortical subarachnoid hemorrhage (SAH), and cortical infarcts. The diagnosis of the gold standard is brain tissue biopsy. However, biopsy is invasive, and as a result, some criteria for the diagnosis of CAA-ri have been based on clinical and radiological findings. Auriel et al^[10]^ revised the diagnostic criteria for CAA in 2016 and proposed CAA-ri diagnostic criteria, which indicated the diagnostic criteria of “possible CAA-ri,” including age ≥40 years, presence of ≥1 of the following clinical features: headache, decrease in consciousness, behavioral change, or focal neurological signs and seizures, and the presentation is not directly attributable to an acute ICH, MRI shows white matter hyperintensity (WMH) lesions that extend to the immediately subcortical white matter. Presence of ≥1 of the following cortico-subcortical hemorrhagic lesions: cerebral macrobleed, cerebral microbleed, or cortical superficial siderosis; absence of neoplastic, infectious, or other cause. Furthermore, the “probable CAA-ri” encompasses asymmetric WMH lesions that are not attributable to previous ICH. Retrospective analyses have demonstrated that a reliable diagnosis of CAA-ri can be achieved based solely on fundamental clinical and neuroradiological assessments, with the diagnosis exhibiting high sensitivity and specificity. Additionally, CSF analysis constitutes an essential component of the CAA-ri diagnostic procedure, as most researchers have observed reduced levels of Aβ42 and Aβ40, along with elevated levels of t-tau, in affected patients.^[11]^ Recent advancements in amyloid positron emission tomography have enhanced our comprehension of Aβ accumulation in cerebral vessel walls, potentially offering diagnostic insights into CAA-ri.^[12]^ Nevertheless, the relevant research remains limited. Initiating early immunosuppressive therapy could potentially ameliorate clinical and imaging outcomes, abbreviate the disease duration, and decrease the recurrence risk in patients with CAA-ri.^[13]^

The patient, a 67-year-old male, presented with an acute onset of cognitive deficits, headaches, and psychobehavioral abnormalities. SWI/FLAIR sequences revealed multiple foci of cortical microhemorrhages, hemosiderin deposition, and asymmetric WMH lesions. Additionally, cortical SAHs were identified on CT and SWI sequences, with no evidence of tumors, infections, or immune abnormalities. CSF analysis indicated a decrease in Aβ and an increase in Tau protein levels. Consequently, a diagnosis of “probable cerebral amyloid angiopathy-related inflammation (CAA-ri)” was initially considered.

CAA-ri is highly heterogeneous clinically. The most common clinical manifestation is presumed to be acute or subacute cognitive impairment, followed by TFNEs, headaches, and seizures. WMH lesions and ICH are by far the most prevalent neuroimaging findings. CAA with vascular inflammation was first described in a patient with AD in 1974,^[14]^ but the mechanism is currently unknown. Three prevailing hypotheses have been proposed^[15]^: the coexistence of Aβ deposition and vascular inflammation suggests a lack of correlation between the two; inflammation may promote the deposition of Aβ within the intracranial vessel walls; and inflammation may result from an autoimmune response to Aβ deposition. Presently, the majority of evidence supports the third hypothesis, implicating Aβ as the primary factor. Consequently, therapeutic strategies should prioritize immunosuppression and the clearance of Aβ. Aβ is a small peptide with 39 to 43 amino acid residues generated by endoproteolytic cleavage of the APP, which relies primarily on the cleavage of its N-terminal by β-secretase.^[16]^ Aβ at physiological concentrations can improve the ability of learning and retention, but excessive deposition is the opposite. Aβ undermines the cognitive function mainly based on the following 3 mechanisms: critical biochemical components involved in memory formation, such as phosphatidylinositol 3 kinase (PI3K), can be inhibited by excessive accumulations of Aβ. Aβ contributes to mitochondrial fragmentation and synaptic loss, while dysfunctional mitochondria may exacerbate cognitive decline by increasing the accumulation of Aβ derived from APP. Furthermore, Aβ deposits can induce oxidative damage and impair the function of transporters within the blood-cerebrospinal fluid barrier, resulting in blood-brain barrier dysfunction and a reduction in Aβ efflux from the brain.^[17]^ The primary characteristics of TFNE include stereotyped, transient, and recurrent neurological manifestations such as paresthesia or numbness, which typically resolve within 10 to 30 minutes due to cortical spreading depression. Current understanding suggests that TFNE is anatomically associated with cSAH and cSS. Furthermore, cSS is identified as a risk factor for future ICH and mortality.^[18]^ The etiology of headache in this context may be attributed to inflammatory stimulation of meningeal pain-sensitive structures and cSAH, as well as potential disruptions in cerebrospinal fluid absorption and secretion caused by small-vessel vasculitis. CAA-ri is independently associated with the occurrence of seizures, with additional contributing factors including ICH and cSS. Some researchers have posited that TFNEs may be indicative of epileptic seizures, thereby underscoring the importance of video-EEG and neurophysiological assessments in the evaluation of patients presenting with TFNEs.^[19]^ The APOE ε4 allele remains the sole confirmed genetic risk factor for individuals with CAA-ri, with 34% of patients exhibiting a homozygous ApoE ε4/ε4 genotype.^[20]^ The APOE ε4 allele is hypothesized to enhance amyloid-beta Aβ deposition and exert a pro-inflammatory influence.

High-dose corticosteroids, with or without adjunctive immunosuppressive therapy, have been shown to potentially enhance outcomes in patients with CAA-ri. In the study conducted by Regenhardt et al,^[13]^ 48 patients diagnosed with CAA-ri and who received early immunosuppressive therapy were included in the analysis. The findings indicated that individuals receiving immunosuppressive therapy exhibited superior clinical and radiographic outcomes compared to those who were not treated, as well as a reduced recurrence rate associated with the use of immunosuppressive therapy.^[13]^ A separate study demonstrated that the disease may relapse following the cessation of therapy or during dose reduction; however, immunosuppressive treatment remains effective in such instances.^[21]^ Presently, the most frequently utilized immunosuppressants include high-dose glucocorticoids, with cyclophosphamide, azathioprine, mycophenolate mofetil, methotrexate, and immunoglobulin also being commonly employed. Post-discharge, the patient was monitored on multiple occasions, revealing a progressive enhancement in cognitive function. Additionally, there was no recurrence of headaches or other symptoms, and the patient was able to perform daily activities independently.

## 4. Conclusion

CAA-ri remains a rare disease. We present a case study of “probable cerebral amyloid angiopathy-related inflammation (CAA-ri)” in which immunosuppressive therapy was administered promptly, resulting in significant patient improvement. This case underscores the importance of timely diagnosis and intervention in patients with CAA-ri, as early management of the inflammatory process can significantly influence clinical outcomes. It is important to acknowledge that the absence of a cranial MRI review for this patient constitutes a limitation of the study. Regular imaging assessments should be conducted in patients with CAA-ri to monitor changes in lesions. Notably, there is a paucity of research on CAA-ri, highlighting the need for further investigation in this area. Future research should concentrate on elucidating the pathogenesis, identifying risk factors, and analyzing cerebrospinal fluid and biological markers associated with CAA-ri. It is crucial to determine which patients with CAA are predisposed to developing CAA-ri to enhance preventive strategies. Given that CAA-ri represents an autoimmune inflammatory response aimed at clearing Aβ, further investigation into Aβ clearance mechanisms is warranted. Additionally, the development of a standardized treatment protocol requires continued exploration.

## Acknowledgments

The authors would like to thank Yanhong Dong for her assistance in writing this manuscript.

## Author contributions

**Methodology:** Yao Meng, Lingyu Li.

**Writing—original draft:** Yao Meng.

**Formal analysis:** Yining Xiao, Ruohan Sun.

**Resources:** Yining Xiao.

**Writing—review & editing:** Yining Xiao.

**Supervision:** Yanhong Dong.
